# The Multidisciplinary Support To Access living donor Kidney Transplant (MuST AKT) intervention: A Pilot Randomized Controlled Trial

**DOI:** 10.3389/ti.2026.15472

**Published:** 2026-02-25

**Authors:** Anne-Marie Selzler, Parastoo Molla Davoodi, Scott Klarenbach, Ngan N. Lam, Terry Smith, Abigail Ackroyd, Ben Vandermeer, Bonnie Corradetti, Aman Dhaliwal, Sharron Ferdinand, Dorothy Ikekekpolor, Gordon Smith, Aminu K. Bello, Kevin Wen, Soroush Shojai

**Affiliations:** 1 Medicine Strategic Clinical Network, Alberta Health Services, AB, Canada; 2 Adult Cystic Fibrosis, Kaye Clinic, Alberta Health Services, Edmonton, AB, Canada; 3 Department of Medicine, University of Alberta, Edmonton, AB, Canada; 4 Department of Medicine, Cumming School of Medicine, University of Calgary, Calgary, AB, Canada; 5 Alberta Kidney Care North, Alberta Health Services, Edmonton, AB, Canada; 6 Transplant Services, University of Alberta Hospital, Alberta Health Services, Edmonton, AB, Canada; 7 Department of Medicine, University of British Columbia, Vancouver, BC, Canada

**Keywords:** kidney transplant, living kidney donation, pilot study, randomized controlled trial, transplant

## Abstract

We evaluated the feasibility and acceptability of the Multidisciplinary Support To Access living donor Kidney Transplant (MuST AKT) intervention, developed to increase living donor kidney transplantation (LDKT). In this pilot randomized controlled trial, we randomly assigned transplant candidates to receive standard care or the MuST AKT intervention, where transplant candidates and their social network addressed barriers to LDKT across four 60–90-minute sessions. Feasibility was assessed by consent/recruitment, retention, study protocol adherence, intervention adherence, and intervention engagement. Acceptability was assessed by questionnaire and post-intervention interviews. The recruitment rate was 61% (43/71), with 38 participants randomized 1:1. Among intervention participants, 1 was excluded for not meeting study criteria prior to start. Among those that started (18), 100% completed 1 session, 94% completed 2 sessions, 83% completed 3 sessions, and 56% completed all 4 sessions. The intervention was delivered in 71 days (mean), shorter than anticipated. The intervention participants reported increased confidence for communicating about LDKT from pre-to post-intervention, and all recommended MuST AKT to their peers. Intervention participants and invitees from their social network described a positive experience and provided recommendations for improvement. The MuST AKT intervention is feasible with minor modifications and acceptable to transplant candidates and their social network.

## Graphical Abstract

**Figure:**
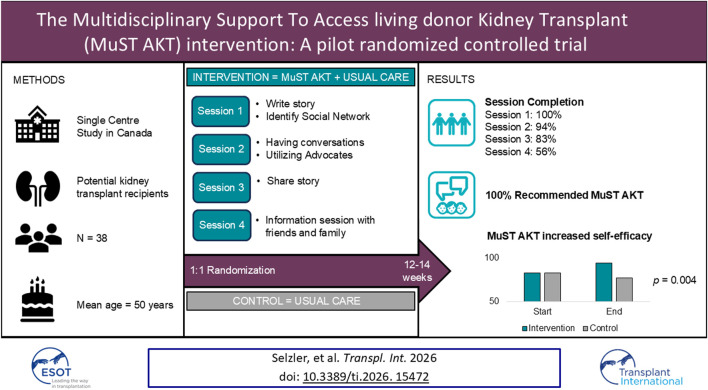

## Introduction

Compared with deceased donor kidney transplantation, living donor kidney transplant (LDKT) provides longer patient and graft survival [1] and shorter wait-time to transplantation [2]. Pre-emptive LDKT can prevent the need for dialysis and reduce the shortage of organs (kidney) for transplantation. However, LDKT is underutilized and many potential transplant recipients report being hesitant to approach potential living kidney donors due to fear, guilt, lack of knowledge, and lack of confidence to communicate effectively about living kidney donation [3–9].

Several interventions have been developed to increase LDKT. The most effective interventions are individually and culturally tailored, and designed to increase knowledge about LDKT and facilitate communication between potential recipients and their social network [10–13]. These interventions have led to increased potential donor evaluations [12, 14–17]. In a scoping review, similar interventions have been found to increase the number of potential donors contacting transplant programs by 40%–50%, the number of potential donors evaluated by 25%–47%, and the number of LDKT by 34% [18].

To address patient barriers to LDKT in our healthcare jurisdiction, we collaboratively developed the Multidisciplinary Support to Access living donor Kidney Transplant (MuST AKT) intervention [19] with patients, healthcare providers, and administrators, with the long-term goal of increasing LDKTs. Similar to previous successful interventions, MuST AKT targets transplant candidates and their social network to address knowledge and communication barriers to LDKT. In our design of MuST AKT, we applied the COM-B model [20] of behavior change to address barriers to LDKT and specifically enhance transplant candidates’ motivation (M) and capability (C) to communicate about LDKT and support them with the creation of opportunities (O) for communication. Consistent with recommendations for developing, evaluating, and implementing complex interventions [21, 22], we first conducted a pilot study with the objective of assessing the feasibility and acceptability of the MuST AKT intervention.

## Materials and Methods

### Study Design and Setting

We conducted a pilot parallel RCT with a nested qualitative study following qualitative description methodology [23, 24]. In this study we adopted a pragmatic worldview, acknowledging multiple realities (ontology) and valuing diverse approaches to knowledge (epistemology) to understand and evaluate a program to support people with their personal search for a living kidney donor. This study was conducted at a single regional academic transplant referral center with a catchment area population of >2 million. Participants were randomly assigned 1:1 to either the experimental (MuST AKT) or control (standard care) arm. Institutional review board approval was obtained from the University of Alberta Health Research Ethics Board–Health Panel and Northern Alberta Clinical Trials and Research Centre (Pro00097902). The trial was registered with ClinicalTrials.gov (NCT04666545) and the CONSORT extension for pilot and feasibility trials [25] was used for reporting. Study enrollment occurred between May 2021 and February 2022. A comprehensive description of the MuST AKT intervention and study protocol is published elsewhere [19].

### Participant Recruitment and Procedures

#### Patient Participants

Participants were recruited from the standard care ‘Introduction to Kidney Transplant’ education class or transplant waitlist at the University of Alberta Hospital. The inclusion criteria were referred or approved for kidney transplantation, aged between 18 and 75 years old, and English-speaking. The exclusion criteria were having a potential living kidney donor who contacted the living donor program, previously received a solid organ transplant, a candidate for a multi-organ transplant, scored <19 on the Rapid Estimate of Adult Literacy in Medicine (REALM-66) – indicating illiteracy in English [26] or scored >20 on the Stanford Integrated Psychosocial Assessment for Transplant (SIPAT) – indicating less than a good candidate for transplant [27]. The SIPAT is a standard assessment tool used at our transplant center and was conducted to support the inclusion of patients referred for transplant who were likely to be approved. No patients already approved for transplant were excluded by the SIPAT. A social worker conducted the REALM-66 and SIPAT using a virtual communications platform. The project manager obtained written informed consent electronically via the REDCap database [28]. Those who declined to participate were invited to take part in an interview to expand on their reasons for declining.

Subsequently, participants completed a baseline questionnaire (socio-demographics and self-efficacy) over the telephone with the project manager, who then randomized participants in the REDCap database [28] using predetermined randomly generated permuted blocks of 4 and 6 created by a statistician in Stata/MP 17.0 [29]. All participants completed a post-study questionnaire (acceptability and self-efficacy) after their intervention or time-control (12–14 weeks following enrollment), as appropriate. Participants in the experimental (MuST AKT) arm were invited to participate in a post-intervention interview.

#### Invitees of Patient Participants

All English-speaking individuals who attended the final MuST AKT intervention session at the invitation of the patient participant were invited to participate in a qualitative interview after the session. An independent qualitative researcher obtained written informed consent electronically via the REDCap database [28].

### Interventions

Participants in the experimental arm received the MuST AKT intervention plus standard of care, whereas participants in the control arm received standard of care only.

#### Multidiscipinary Support To Access living donor Kidney Transplant (MuST AKT)

**FIGURE 1:**
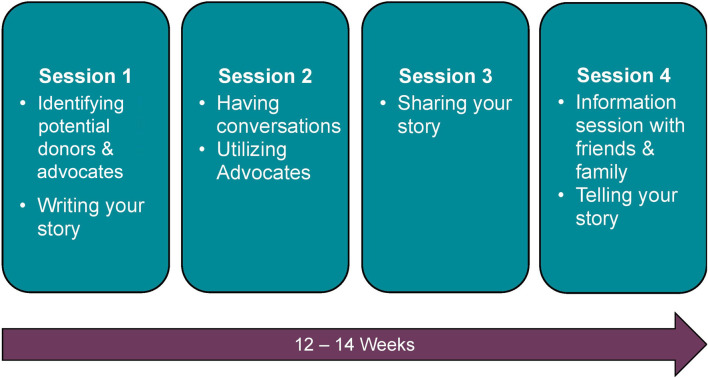
Outline of the MuST AKT intervention sessions.

MuST AKT is a person-centered evidence-informed intervention designed to address barriers to LDKT, including lack of knowledge about LDKT and the process for potential donors, difficulty communicating about LDKT, and lack of social support, which were previously identified by transplant candidates in our healthcare jurisdiction [30]. The intervention was co-designed with patients and family advisors with lived experience of chronic kidney disease and kidney transplant, nephrologists and healthcare providers with expertise in kidney transplant, behavioural scientists, and social media experts from the Kidney Foundation of Canada–Alberta branches. Intervention strategies outlined in the COM-B model [20], including education (e.g., providing information, including how to use social media safely and appropriately), training (e.g., activities, including identifying donors and advocates), enablement (e.g., solution-focused interviewing to identify and overcome barriers to having conversations about LDKT), and modelling (e.g., role-playing on how to initiate and sustain conservations about LDKT) were applied to address these barriers. The sessions included structured activities and flexible time to support patients with identifying and overcoming their own personal barriers to LDKT. A complete description of the MuST AKT intervention has been published elsewhere [19]. In short, the MuST AKT intervention consists of an introductory session followed by 4 intervention sessions delivered over 12–14 weeks (approximately 1 session every 3 weeks), each ranging from 60–90 minutes in length, to identify and address barriers to LDKT. Figure 1 outlines the main topics of each intervention session. All sessions were one-on-one with a behavioral scientist or a social worker, with the exception of the final session, in which the transplant candidate invited individuals from their social network to attend an information session on kidney disease and living kidney donation. This session also included an opportunity for participants to share their kidney disease and LDKT journey with invitees, and a question-and-answer period for which a transplant nephrologist was in attendance. The intervention was designed to be delivered in-person or virtually. Due to the COVID-19 pandemic, all sessions were conducted virtually.

Participants received reminder phone calls or emails 1–2 days prior to their scheduled sessions to promote session attendance. At their own request, participants could discontinue their participation in the intervention or withdraw from the study (i.e., no further data collection). Participants were withdrawn from the study if they did not respond to the study team after 3 contact attempts (as per HREB guidelines). Reasons for discontinuation and withdrawal were tracked.

#### Standard of Care

At our kidney transplant program, standard of care includes a standardized virtual group transplant education class (2 hours), arrangement of the required tests and consultations for transplant evaluation, and medical assessment for transplant suitability by a social worker, transplant nephrologist, and surgeon. Prior to the virtual education class, patients were provided with resources on how to use the virtual communication platform and one-on-one technical support to access the class. The transplant education is delivered by a nurse coordinator with topics including benefits and risks of kidney transplantation, living kidney donation, the kidney paired donation program, deceased kidney donation, evaluation and waitlist procedures, transplant surgery, post-transplant medicines and recovery. Additionally, patients were given a link to a kidney transplant educational website (https://myhealth.alberta.ca/KidneyTransplant) and access to the teaching materials. These resources were provided to all patients prior to their participation in the study, with no additional internal resources provided as part of standard care during the study period. During their transplant work-up, patients are supported by a transplant nephrologist, nurse coordinator, and social worker.

### Outcomes

Our joint primary outcomes were feasibility and acceptability, with feasibility assessed by consent/recruitment, retention, adherence to study protocol (fidelity), adherence to intervention, and engagement in intervention. Acceptability was assessed by questionnaire and interview (Table 1; Supplementary Material for guides and questions). A secured REDCap database [28] was used to collect and store survey and study process data. NVivo-12 software was used to organize and store qualitative data [32].

**TABLE 1:** Feasibility and acceptability (questionnaire) outcomes.

Outcome	Details
Consent/recruitment	Consent rate (n^consented^/n^approached^) for patient participants and invitees of session 4 was tracked by consent logs, with reasons identified by structured survey
Retention	The number of patient participants that withdrew from the study and the number that were discontinued (session stoppage) were tracked by logs, with reasons identified by structured interview/survey
Adherence to study protocol (fidelity)	The duration of the intervention (from session 1–4) was tracked by logs of session attendance dates
Adherence to intervention	Attendance rate (n^attended^/n^allocated^ to MuST AKT) for each session, and the rate of sessions rescheduled combined across all 4 sessions (n^sessions rescheduled^/n^total possible sessions^) were tracked by attendance logs. Reasons for rescheduling sessions were identified by structured surveys
Engagement in intervention	Intervention engagement was measured by logs tracking completion of each step/component of the MuST AKT intervention (fidelity), the number of participants who wrote their stories at the end of MuST AKT session 1, the number of potential donors and advocates identified during session 1 of the MuST AKT intervention, the number of participants who found at least one potential advocate at the end of the MuST AKT intervention (12–14 weeks), and the number of participants who started conversation with at least one potential donor by the end of the last MuST AKT session
Acceptability (questionnaire)	A post-study questionnaire was conducted with patient participants 1–2 weeks after completing the MuST AKT intervention or time control. ‘Recommendation of MuST AKT to others’ and ‘perception of intervention effectiveness’ were measured using likert scales from 1 (completely disagree) to 7 (completely agree); and ‘self-efficacy for finding a living kidney donor’ and ‘self-efficacy for communicating about LDKT’ were measured on a scale from 0 (not at all confident) to 100 (completely confident). Self-efficacy was also assessed prior to randomization. Self-efficacy was assessed by items designed specifically for this study. These items were developed to assess the specific types of self-efficacy we were trying to impact with our intervention (i.e., confidence for communicating about LDKT, and confidence for finding a living kidney donor). Importantly, these items adhere to the theoretical and measurement guidelines outlined by Bandura [31].

#### Acceptability (Interview)

The interview methodology was guided by qualitative description [24], where language is viewed as a vehicle of communication rather than an interpretive structure to be deciphered. In this approach the researcher seeks to present an accurate account of experiences using everyday language [24]. The interview guide was informed by the COM-B model [20] and written in everyday language to be consistent with qualitative description methodology. Semi-structured interviews were conducted over the telephone or virtual communication platform with (i) participants allocated to receive the MuST AKT intervention and (ii) their invitees who attended session 4 of the MuST AKT intervention. The purpose of the interviews was to explore their perspectives of the intervention sessions attended (including content), and to receive recommendations for improvement of the intervention. The interviewers were not healthcare professionals, had no pre-existing relationship with the participants, and were independent of the study team. The interviews were digitally recorded and transcribed clean verbatim. MuST AKT participant interviews ranged from 25–55 min and the invitee interviews ranged from 15–30 min.

#### Demographics, Social, and Clinical Characteristics

Participant age, gender, ethnicity, education, employment status, income, household status, dialysis (yes/no), duration of dialysis, and cause of kidney disease were collected from electronic medical databases or structured survey.

#### Changes to Trial Assessments

The following efficacy outcomes were collected in monthly reports and validated by comparing reports to individual electronic charts: proportion of participants with at least 1 potential donor who (i) contacts living donor services, (ii) starts evaluation, and (iii) completes evaluation; along with (iv) the proportion who receive a LDKT. After trial commencement but before completion, we decided to postpone the analysis of efficacy outcomes in order to pool the pilot data with the subsequent larger definitive RCT (funding secured during pilot for definitive RCT with power more favorable with pooling). The timeframe for collecting LDKTs was changed from 12 to 24 months in the pilot study to match the definitive RCT timeframe (clinicaltrials.gov: NCT04666545).

We planned to conduct interviews with people who declined to participate in the study to better understand their reasons for declining; however, no one consented to an interview.

### Sample Size

Prior to the decision to postpone the analysis of the efficacy outcomes in this pilot study and pool the data with the definitive RCT data, we chose a sample size of 38 participants (19 per group), which is consistent with recommendations for pilot studies [33, 34]. Sample size calculations for the efficacy data are described elsewhere [19].

### Blinding

It was not possible to blind the participants or the research team as to which intervention the participants received, although intervention allocation was not revealed until the participant was enrolled in the study and completed the baseline questionnaire. The quantitative and qualitative data analyses were conducted by a statistician and a qualitative research team, respectively, not involved in the conduct of the study.

### Statistical Method

#### Quantitative Analysis

All analyses were conducted in Stata/MP 17.0 (www.stata.com) following an intention-to-treat approach. Descriptive statistics were conducted to summarize the data. T-tests were conducted to compare the difference in pre- and post-intervention self-efficacy (acceptability) scores across treatment arms, with *p* < 0.05 considered statistically significant.

#### Qualitative Design and Analysis

The interview data was analyzed using thematic analysis [35]. Two researchers read through the transcripts several times, while identifying meaningful information related to the research objectives. Next, key words or statements were selected from the data and then grouped into codes. A coding framework was established based on the COM-B informed interview guides and additional codes that emerged from the data. Including an inductive coding approach allowed us to stay close to the recounting of events as described by participants and interpret events beyond our theoretical disposition, consistent with qualitative description [24]. The coding framework was validated by two coders, with any disagreements resolved through discussion. The codes were revised and reviewed for each interview and then grouped into common themes. The themes were reviewed and compared across the interviews. Then, the themes were described and linked with quotes.

## Results

### Recruitment, Enrollment, and Retention

**FIGURE 2:**
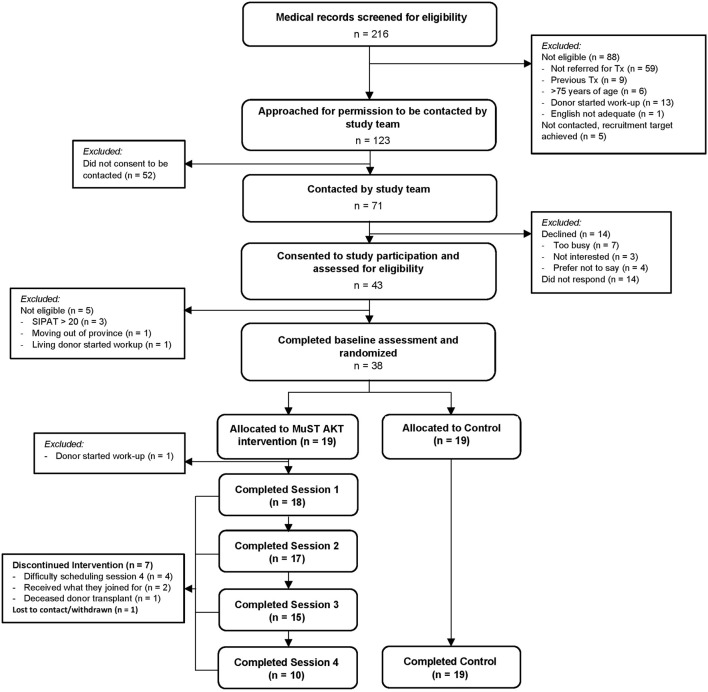
Participant Flow Diagram for screening, eligibility, enrollment, randomization, and session completion.

Figure 2 displays the complete study flow with reasons for exclusion and discontinuation. In short, 43 of 71 transplant candidates consented (61%). The most common reason for non-consent was non-response after 3 contact attempts (n = 14). Of the 43 consenting participants, 5 were ineligible and excluded, and 38 were randomized 1:1 to the MuST AKT intervention or standard care arms. Of the 19 participants allocated to the MuST AKT arm, 1 did not receive the intervention as they were excluded after randomization (and from analysis) for having a living donor in evaluation. Of the 18 who started the intervention, 10 fully completed, and 8 partially completed (n = 7 discontinued, 1 lost-to contact/withdrew). Reasons for discontinuation include difficulty scheduling Session 4 (n = 4), received what they joined for–started communicating with social network about LDKT (n = 2), and received a deceased donor kidney transplant (n = 1). In the MuST AKT arm, 17 participants completed assessments (10 who completed the intervention, 7 who partially completed). In the standard of care arm, all 19 participants completed the assessments at the time control (12–14 weeks after enrollment).

### Sociodemographic and Clinical Characteristics

Baseline characteristics of participants in the MuST AKT and control arms were similar, although, there was a greater representation of non-Caucasian and employed participants in the MuST AKT arm compared to the control arm (Table 2). Among the participants who chose to discontinue the intervention (n = 7), 86% (6/7) were male, 71% (5/7) were non-Caucasian, 86% (6/7) had at least some-post secondary or trade certification, 71% (5/7) were employed, 57% (4/7) had income > $90,000CAD, and 86% (6/7) lived with someone, 57% (4/7) lived with their spouse.

**TABLE 2:** Participant sociodemographic and clinical characteristics at enrollment.

Characteristics	Experimental arm (n = 19)	Control arm (n = 19)
Age, years, mean (SD)	49.8 (16.2)	50.9 (15.8)
Gender, female, n (%)	6 (31.6)	8 (42.1)
Ethnicity^a^, n (%)		
Caucasian	7 (36.8)	12 (63.2)
Indigenous	6 (31.6)	2 (10.5)
Non-Caucasian nor indigenous	7 (36.8)	5 (26.3)
Highest level of education, n (%)		
University Degree	7 (36.8)	2 (10.5)
College Diploma	5 (26.3)	4 (21.1)
Trade certification/some post- secondary	4 (21.1)	7 (36.8)
High school Diploma	3 (15.8)	4 (21.1)
Elementary/some high school	0 (0.0)	2 (10.5)
Employment status, n (%)		
Full-time	8 (42.1)	5 (26.3)
Part-time or casual	4 (21.1)	3 (15.8)
Not employed	1 (5.3)	3 (15.8)
Student	1 (5.3)	0 (0.0)
Disability	2 (10.5)	4 (21.1)
Retired	3 (15.8)	4 (21.1)
Household income, Canadian dollars, n (%)		
$0–49,999	5 (26.3)	2 (10.5)
$50,000-$99,999	4 (21.1)	9 (47.4)
$100,000-$149,999	1 (5.3)	2 (10.5)
≥$150,000	7 (36.8)	1 (5.3)
Prefer not to say	2 (10.5)	5 (26.3)
Household status, n (%)		
Alone	5 (26.3)	3 (15.8)
Spouse	10 (52.6)	12 (63.2)
Immediate family member	3 (15.8)	4 (21.1)
Friend(s) or roommate(s)	1 (5.3)	0 (0.0)
Dialysis at enrollment, n (%)	10 (52.6)	13 (68.4)
Days on dialysis at enrollment, median (IQR)	139 (67, 307)	151 (114, 456)
Cause of kidney disease		
Diabetic nephropathy	7	5
Glomerulonephritis	7	9
Hypertension	1	1
Congenital	1	0
Polycystic kidney disease	0	1
Thrombotic microangiopathy	1	0
Other	0	1
Unknown	2	2

^a^ One participant in “Treatment Group” identified as both Caucasian and Indigenous.

### Intervention Adherence, Engagement, and Fidelity

**FIGURE 3:**
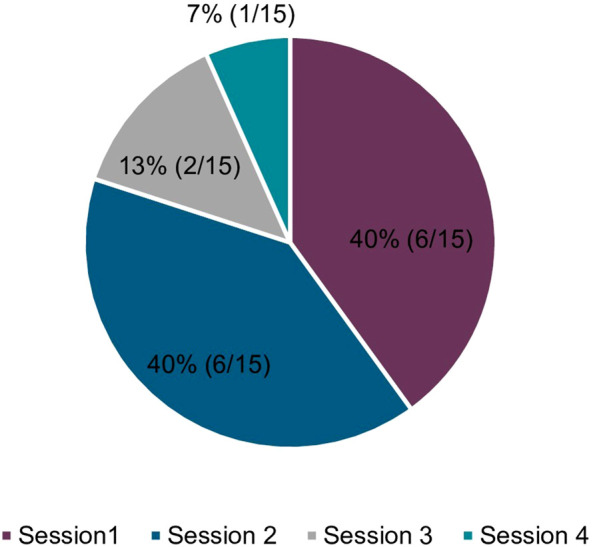
The number of MuST AKT sessions rescheduled (15 total out of possible 60). Reasons for rescheduling included, forgot/mixed up dates (n = 6), unwell/emergent health visit (n = 3), work/school (n = 3), too busy (n = 1), caring for dependent (n = 1), wanted more time before next session (n = 1), facilitator scheduling conflict (n = 1), no friends/family members attended session 4 (n = 1). Multiple reasons for non-attendance were recorded, if applicable.

Session attendance was 100% (18/18) for Session 1 but decreased throughout the sessions, with 94% (17/18) completing the first 2 sessions, 83% (15/18) completing the first 3 sessions, and 56% (10/18) completing all 4 sessions. A total of 25% of sessions were postponed and rescheduled (15/60) mainly as participants forgot/mixed up dates (n = 6) (Figure 3). The planned intervention duration, from session 1 to 4, was 84–98 days (12–14 weeks) and the average (SD) observed intervention duration was 71 (22) days.

Among those that attended, the completion rate across all in-session activities was 76% (142/187), with Session 2 and 4 having the lowest and the highest activity completion rate, respectively (40/68 = 59% and 10/10 = 100%) (Table 3). All participants who attended the first intervention session identified at least one potential advocate and one potential donor by the end of the MuST AKT sessions, with a median (IQR) of 17.5 (8, 25) advocates and 10.5 (7, 19) potential donors identified for each participant.

**TABLE 3:** In-session activity completion rates and reasons for non-completion.

Activities across 4 sessions	Completion rate	Reasons for non-completion
Session 1 activity (n = 18) 1. Write story	18/18 (100%)	​
2. Family tree	18/18 (100%)	​
3. Discussed social and financial challenges & solutions	3/18 (16.7%)	• Session delivered by non-social worker without this expertise (n = 12)• Not enough time (n = 2)• Covered in screening (n = 1)
**Total session 1 activity completion**	**39/54 (72.2%)**	​
Session 2 activity (n = 17)		
1. Create plan for sharing story	3/17 (17.6%)	• Reasons not tracked
2. Short message prepared	9/17 (52.9%)	• Not enough time (n = 3)• Participant declined (n = 3)• Not relevant to participant (n = 2)
3. Discuss & identify best advocates	11/17 (64.7%)	• Not enough time (n = 5)• Participant declined (n = 1)
4. Discuss challenges & solutions to one-on-one conversations	17/17 (100%)	​
**Total session 2 activity completion**	**40/68 (58.8%)**	​
Session 3 activity (n = 15)		
1. Create story	15/15 (100%)	​
2. Discuss challenges & solutions to group conversations	13/15 (86.7%)	• Not enough time (n = 2)
3. Session 3 slideshow reviewed in full (how to create effective messages)	15/15 (100%)	​
**Total session 3 activity completion**	**43/45 (95.6%)**	​
Session 4 activity (n = 10)		
1. Session 4 slideshow reviewed in full (information on kidney disease and LDKT)	10/10 (100%)	​
2. Question and answer period for friends and family members	10/10 (100%)	​
**Total session 4 activity completion**	**20/20 (100%)**	​
Total activity completion across all sessions	**142/187 (76%)**	​

Bold text indicates a cumulative completion rate across each individual session and all sessions combined.

### Acceptability

#### Questionnaire

There were statistically significant differences in self-efficacy for communicating about living kidney donation (*p* = 0.004) and self-efficacy for finding a living kidney donor (*p* < 0.001) between the MuST AKT intervention and control arms from pre- to post-intervention. Participants in the intervention arm reported increased confidence for communicating about living kidney donation and finding a living kidney donor from pre- to post-study, whereas participants in the control arm reported decreased confidence (Table 4). All participants in the intervention arm recommended the program and found it effective.

**TABLE 4:** Participant reported acceptability by study arm.

Acceptability measure	Pre-study		Post study		*p*-value
	Experimental arm (n = 17)	Control arm (n = 19)	Experimental arm (n = 17)	Control arm (n = 19)	
Recommend to others, n (%)	​	​	​	​	​
Completely agree	​	​	15 (88.2%)	​	​
Moderately agree	​	​	1 (5.9%)	​	​
Slightly agree	​	​	1 (5.9%)	​	​
Neither agree nor disagree	​	​	0 (0.0%)	​	​
Slightly disagree	​	​	0 (0.0%)	​	​
Moderately disagree	​	​	0 (0.0%)	​	​
Completely disagree	​	​	0 (0.0%)	​	​
Program was effective, n (%)	​	​	​	​	​
Completely agree	​	​	6 (31.6%)	​	​
Moderately agree	​	​	5 (29.4%)	​	​
Slightly agree	​	​	3 (17.7%)	​	​
Neither agree nor disagree	​	​	2 (11.8%)	​	​
Slightly disagree	​	​	1 (5.9%)	​	​
Moderately disagree	​	​	0 (0.0%)	​	​
Completely disagree	​	​	0 (0.0%)	​	​
Self-efficacy^a^, 0–100, mean (SD)					
Confidence for communicating about LDKT	83 (23)	83 (25)	94 (13)	77 (26)	0.004
Confidence for finding LKD	62 (27)	61 (25)	81 (22)	47 (28)	<0.001

LDKT, living donor kidney transplant; LKD, living kidney donor.

^a^ t-test

#### Nested Qualitative Study

Interviews were conducted with participants in the experimental arm, referred to as “MuST AKT participants” and those who they invited to session 4, referred to as “invitees”. Of the MuST AKT participants, 11 of 19 (58%) completed an interview (8 fully completed intervention, 3 partially completed). Of the invitees, 16 of 89 (18%) agreed to and completed an interview, with an average of 2 invitees per session, representing 7 of 10 (70%) session 4’s. Most MuST AKT participants interviewed were male (6/11 = 55%), whereas most invitees were female (12/16 = 75%).

The report provided by the independent qualitative research team is summarized below [36]. Although the MuST AKT participants and invitees were interviewed with a different focus (the entire program versus one session) and using different interview guides, the data analysis revealed similar themes across both groups. As such, the results are presented together. We distinguish MuST AKT participant and invitee data when necessary. Additionally, similar themes were found across participants who fully and partially completed the intervention, and no distinction is made between these groups.

Participants’ views were understood within 4 main themes and 9 sub-themes: (1) Intervention expectations and experience (exceeded expectations, positive & informative experience), (2) MuST AKT sessions (content, sharing my story, social media, logistics), (3) Intervention facilitators, and (4) Intervention Effectiveness (intervention impact, encouraged to be an advocate, encouraged to be a donor). Representative quotes for each theme and sub-theme are presented in Table 5 and referred to in the text below (E.g., quote 1 [Q1], etc).

**TABLE 5:** Themes, sub-themes, and representative quotes from participants.

Themes	Sub-themes	Representative sentence
Intervention expectation and experience	Expectations exceeded	“It was way beyond my expectations. [….] it almost felt like it was personalized. [MuST AKT facilitator 1] was just fantastic. […] I’m not saying I cannot do things like by myself, but it is really hard to do some of these things like […] writing the story. Even though it sounds simple, it is not that simple.” – MuST AKT participant (Q1) “Obviously it is a pretty serious issue, but even still, you do hear a lot of negative things more so than positive about the state of our healthcare system. To see two healthcare professionals come together for one person and do a 2-h info session on a zoom call in an evening, yeah it was really really impressive.” – Friend and family (Q2)
	Positive & informative experience	“No, I just enjoyed it so much you would not believe. It was awesome. It was great. I learnt so much. I learnt to open up to friends and all that. I have good word for it.” – MuST AKT participant (Q3) “I did not expect that it would be – I guess in the end it was so educational, such good information. I thought wow, we were not just looking at slides, we had professional people answering our questions and it went really well I thought.” – Friend and family (Q4)
MuST AKT sessions	Content	“[…] I really appreciated the graphics and the slides and being able to see what [MA facilitator 1] was talking about and coming away with a clear understanding of where [MA participant] is going to be going as [they] continue on this journey.” – Friend and family (Q5) “[…] with the specialist they had given me a binder. That was their extent of their education. When I compare my experience with the medical team I had and with what [MuST AKT facilitator 1] and that specialist provided, it was quite a difference. The session was much more informative and personal.” – MuST AKT participant (Q6) “ […] I did have a lot of questions so I was curious to hear from professionals […]. Google can be a bit daunting just because there is so much information, so it was nice to hear from people who are actively working with [MuST AKT participant] about the kidney transplant process.” – Friends and family (Q7) “Just put more emphasis on what an advocate can do. [There is more emphasis] towards a living donor […] which is great, but if you could also add to that advocacy part that would be beneficial.” – MuST AKT participant (Q8)
​	Sharing my story	“It helped me to talk about it because I was not ready to talk to anyone even my family, even my friends because I felt not guilty, it is hard to explain but I did not want to bother them with that. It was my burden. I’m taking care of it, but [they] helped me go through to open up to [my friends and family].” – MuST AKT participant (Q9) “It walked me through this is something do not think I’d even be able to get this far on my own because there was always that reluctance. I did not know how to go about it. It was there providing the support that I needed and helped me get through all these hurdles” – MuST AKT participant (Q10)
​	Social media	“Yeah so if I post something and a couple of my friends will share it and let’s say they have 500 friends on their friend list then maybe 10% will read it or curious and search it. That’s 50 people that will find out about it. It is just ongoing right.” – MuST AKT participant (Q11) “My [sibling] was a good help. When I sent [them] my story, [they] said can I share that? I said […] share it to the family and friends first. Don’t put it on Facebook […]. [They] sent everything to everyone the nieces, the nephews. Everybody was calling me […]. It was like social media.” – MuST AKT participant (Q12)
​	Logistics	“I think it was scheduled pretty good, […] I was not overwhelmed. At times it was overwhelming because it seems I got a lot of appointments and busy, busy, busy.” – MuST AKT participant (Q13) “If you take a [religious] household where maybe the kids are not religious, they drink, they smoke and they obviously cannot say that to their parents. […] that was really important to ask that question, but I obviously could not go into detail.” (Q14)
Intervention facilitators	​	“I think we lucked out with the people that were helping with the session. They were very personable and quite emotionally intelligent, responding to cues and humor […] I think for this program to be successful, you really have to strike it with the people hosting because [they] are 80% of it. People will participate if dealing with someone more personable.” – Friend and family (Q15) “I felt that [they were] with me and understood me very empathetic.” – MuST AKT participant (Q16)
Intervention effectiveness	Intervention impact	“I think it is very important. I would hate for the program to not continue because in my opinion it improved our life and if it improves one or two other people, which I know it’ll improve more, it’s beneficial. It should happen.” (Q17)
	Encouraged to be an advocates	“I think ultimately as a family member you do have to be an advocate regardless, but I think it made it easier. […] it refines your knowledge a bit on the actual issues.” – Friend and family (Q18) “It just really reassured us that this was something that we should be doing. We were supporting [them] personally, but not going out and letting other people know here we are, we are in this situation and we need that kidney.” – Friend and family (Q19)
	Encouraged to be a donor	“I mean yes and no. It is very tough. The session definitely made me want to actually reach out to the next level of inquiry so I do plan on reaching out and calling that number and setting up a meeting to see what the first steps would be to run some tests. I do not know if it actually made me more likely to go through with it, but it has definitely made me more likely to go through the first steps of it.” – Friend and family (Q20) “It gave me the information that would be needed to contemplate that decision with a little bit more clarity and a more informed decision.” – Friend and family (Q21)

##### Intervention Expectations and Experience

Most MuST AKT participants did not have specific expectations of the intervention, whereas the invitees’ expectations of session 4 varied according to what they were told by the MuST AKT participant (e.g., no specific expectation, a campaign to promote donation, learn about donation process). Regardless of expectations, MuST AKT participants and invitees reported a positive experience, which exceeded their expectations (Q1 and Q2). MuST AKT participants found the intervention informative, provided personalized support, and helped and guided them to share their story (Q1 and Q3). Invitees reported that session 4 was well facilitated and informative (Q4).

##### MuST AKT Sessions

MuST AKT participants and invitees reported that they valued learning clear and accurate information (Q5). MuST AKT participants emphasized the value of this approach over previous clinical interactions and felt more knowledgeable about their disease and circumstances (Q6). Invitees valued learning from healthcare professionals working with the MuST AKT participant compared to the internet (Q7). Both MuST AKT participants and invitees wanted a stronger focus on advocacy in the sessions (Q8).

MuST AKT participants reported that the intervention provided the support they needed to process their circumstances and emotions and share their story (Q9 and Q10). Most (10/11) reported sharing their story with others, with 50% (5/10) sharing their own story through social media, 20% (2/10) planning to share it on social media in the future, and 40% (4/10) asking others to share their story through social media. Sharing their story through social media and through advocates were considered important methods for communicating with their social network (Q11 and Q12). One-on-one conversations was another common way that participants shared their story.

MuST AKT participants were satisfied with the scheduling and logistics of the sessions (Q13). Invitees recommended having time without the MuST AKT participant present in session 4 to ask sensitive questions (e.g., drug and alcohol use) (Q14). See Table 6 for a list of other logistical recommendations to improve MuST AKT sessions.

**TABLE 6:** Logistical recommendations to improve MuST AKT sessions as provided by MuST AKT participants and friends and family.

MuST AKT participant recommendation (all sessions)	Friends and family recommendations (session 4 only)
• Incorporate more visuals in presentations	• Provide an agenda, session expectations, and session length before the meeting
• Record the sessions so they could share with others	• Clearly state the goal of the session at the beginning
• Have nephrologist attend session(s) to answer medical questions	• Provide another session for friends and family
• Provide more sessions to get more information and support	• Have the option to attend in person
• Provide additional friends and family session to provide more opportunity for them to ask questions	• Include more pauses and opportunities for discussion throughout the presentation
​	• Provide assistance for using zoom
​	• Include more resources after the session (e.g., how to share story and create effective messages, information discussed during the session)

##### Intervention Facilitators

Both MuST AKT participants and invitees enjoyed the session facilitators and felt that the qualities of the facilitators (e.g., empathetic, emotionally intelligent, kind, personable) were critical to the success of the intervention (Q15 and Q16).

##### Intervention Effectiveness

MuST AKT participants and invitees agreed that the program was valuable and important to continue (Q17). Invitees reported that session 4 encouraged and empowered them to be an advocate (Q18 and Q19). Some invitees reported that they were motivated to start the evaluation process amidst their uncertainty about donating, but (Q20) noted that it was helpful to have accurate information to help them make a decision (Q21).

## Discussion

The results of this pilot parallel RCT demonstrate feasibility of the MuST AKT intervention as assessed by outcomes of recruitment, intervention fidelity, adherence and engagement in the intervention, and acceptability of the intervention, with modifications needed to improve participant retention (completion of all 4 sessions) before further evaluation. The participant recruitment rate projects a recruitment timeline of 16–17 months for the definitive RCT (64 total participants/3.8 pilot participants per month), which is feasible for timely achievement of broader project commitments. This recruitment rate is satisfactory (61%) given the long-term and intensive commitment required for the intervention and is within the range of what has been reported in similar studies [14, 15]. Most intervention sessions were well attended (3 of 4 sessions with >80% attendance) and there were an acceptable number of intervention sessions rescheduled (15/60 = 25%), which was similar to what is observed for outpatient appointments at our center. We received positive feedback on the content, format, and delivery of the intervention, and had positive accounts of participant reported outcomes. The total intervention duration was shorter than anticipated, which bodes well for future implementation.

To the best of our knowledge, this is the first intervention developed to address barriers to LDKT that was delivered exclusively over a virtual communications platform. Previous similar interventions have included in-person visits between patients, healthcare providers, and the patient’s social network [12, 14–17]. Although the virtual format was implemented out of necessity of the COVID-19 pandemic, a benefit of this format was that we were able to improve healthcare access by reaching patients across a large geographical area, many of whom would have been required to travel multiple hours to attend in-person visits posing a significant barrier to participation. Previous research has shown that virtual modalities are often preferred by patients for non-urgent care due to prompt appointments and convenience, although care should be taken to consider privacy, technology, and connectivity to maintain equity and quality of service [37]. We found that the virtual format was well accepted by both MuST AKT participants and invitees. While some challenges to using the virtual communication platform was noted by the facilitators (e.g., no audio), all challenges were able to be remedied before or during the session. We used the same secure virtual communication platform as the standard of care transplant education class at our health care center. Thus, prior patient experience with the virtual platform may have contributed to few participant challenges.

The finding that patients receiving the MuST AKT intervention reported stronger self-efficacy/confidence for communicating about LDKT after participating is an important benefit. While encouraging, this finding is preliminary, and will be re-evaluated in a definitive RCT. From the lens of the COM-B model, MuST AKT increased motivation to communicate about LDKT, making it more likely that such communication will take place, which is a critical and necessary step to receiving a LDKT. Previous interventions that were found to increase LDKTs and surrogate outcomes [10–12, 14–17] used communication with one’s social network as a core component. Furthermore, self-efficacy has been shown to be a strong predictor of health behaviors across a variety of settings [38] and is associated with readiness to pursue LDKT [39].

Beyond increasing motivation for communicating about living kidney donation, qualitative reports indicate that MuST AKT increased key capabilities of transplant candidates related to finding a living donor, including story writing and sharing, and provided an opportunity (session 4) for communication about living kidney donation that was valued by both transplant candidates and their invitees. As a result of their participation in MuST AKT session 4, some invitees indicated they were encouraged to be advocates and/or living donors. While these are subjective reports, and not objective accounts of donor behaviour, the results provide support for our behaviour change approach guided by the COM-B model [20].

After a multi-session formal review of the results with the study team, intervention facilitators, and patient advisors, minor modifications to the intervention will be made to address participant retention and optimize the intervention before further evaluation (Table 7). We observed a moderate completion rate of all four MuST AKT sessions, which was similar to the TALK intervention [14], but lower than other interventions [15, 17]. The participants who chose to discontinue the MuST AKT intervention were predominantly non-Caucasian males currently employed and living with others. While we did not evaluate the role that these sociodemographic factors played in intervention completion, other studies have also found being male [40] and employed [41] led to lower-rates of health program completion. Based on participant report, almost half (4/9) of the participants who discontinued from this study did so for positive reasons (e.g., began communicating with their social network about LDKT or received deceased donor transplant). Of the remaining discontinuations, participants cited difficulty scheduling session 4 as the most common reason, suggesting this is a key facet to address. Scheduling session 4 with friends and family may present added logistical challenges for employed individuals. While difficulty scheduling session 4 may in part be logistical, the qualitative accounts highlight the emotional fortitude it takes to initiate communication about LDKT. In the definitive RCT, participants will be encouraged to invite a support person/advocate to the earlier intervention sessions to provide emotional and logistical support with arranging session 4, as previous research has found utilizing advocates or “donor champions” to be effective for initiating LDKT conversations and reducing communication barriers to LDKT [42]. We will also include resources for the intervention facilitators to better support patients who experience emotional challenges (e.g., fear, guilt) to communicating about LDKT, including how to utilize the narrative or story from session 1 to address patient challenges. This approach is consistent with techniques of Narrative Therapy [43], which has shown promise for addressing emotional challenges in medicine [44, 45].

**TABLE 7:** Forthcoming modifications to the Multidisciplinary Support To Access living donor Kidney Transplant (MuST AKT) intervention.

Identified area of improvement	Modification
Retention/Intervention adherence – difficulty getting participants to schedule and commit to session 4	• Provide more information in advance about what to expect in session 4 • During session 3, discuss barriers to scheduling session 4 and help identify solutions • Encourage participants to bring a support person/advocate to at least one intervention session (1–3) who could help arrange session 4
Intervention Fidelity/Engagement in intervention – unable to complete all session 1 and session 2 activities in the allotted time • Session 1: social and financial challenges • Session 2: create plan to share story • Session 2: prepare short message • Session 2: discuss and identify best advocates • Session 2&3: discuss challenges and solutions to one-on-one and group conversations	• Discussion of these challenges begins when screening potential participants for the study. Move in depth discussion of social and financial challenges from session 1 to session 2 and 3 • Allow flexibility for facilitator to carry over discussion from session 2 to session 3, if required • Remove – covered in session 3 • Move description and discussion of advocates from session 2 to session 1 • Allow flexibility for facilitator to carry over discussion from session 2 to session 3, if required • Allow more participant generated discussion of LDKT barriers, including barriers that fall outside of ‘having conversations’ such as other social and financial barriers
Acceptability – participant recommended improvements to the sessions • Session 4: Invitation to friends and family • Session 4: Introduction • Session 4: Advocacy • Session 4: Question and answer period • All sessions: Approach to presenting information • Have nephrologist attend session(s) • More sessions	• Remind participants to send out standardized invitation to friends and family with session details. Describe why this is important • Clearly state the goals of the session at the beginning and provide assistance with using zoom, if required • Include additional information and discussion about how to be an advocate • Provide an opportunity for friends and family to ask questions about LDKT without the participant present • Provide more opportunity for pauses and discussion throughout and reduce amount of time presenting • If presentation is required, incorporate more visuals • Develop narrated PowerPoint slides of key session materials so that session content can be shared with friends and family and reflected on afterwards • Not feasible to have nephrologist attend • Ensure training of facilitators so that they are able to answer medical questions • Develop a FAQ document that is vetted by nephrologists and clinicians • Not feasible to include more sessions • Determine what specifically participants and friends and family members would like included if an extra session were to be available. Ask detailed follow-up questions during interviews for the definitive RCT.
Facilitator recommended improvement • Use narrative from session 1 to help address emotion-related barriers (e.g., fear)	• Utilize the narrative or ‘write your story’ activity from session 1 to help address emotional barriers to LDKT. • Develop materials for facilitators to better guide and incorporate the use of narrative in sessions 2 & 3

LDKT = living donor kidney transplantation; FAQ = frequently asked questions; RCT = randomized controlled trial.

Our approach to the MuST AKT initiative to increase LDKT in our healthcare jurisdiction aligns with continuous quality improvement processes, which are integral for ensuring quality in healthcare [46]. Delivery of a process of care/education, by its very nature, slowly changes over time. This adaptive approach ensures responsiveness to patient needs and jurisdictional circumstance.

Our findings should be interpreted within the following contexts and limitations. This pilot RCT was conducted virtually at a single transplant center in a Canadian city with English speaking participants, although there was representation from non-Caucasian racial groups and other groups disadvantaged for transplant including women, those with lower socioeconomic status, and non-urban dwellers. If the MuST AKT intervention is adopted as part of standard care, we will continue to work with our healthcare organization to ensure the intervention is accessible and comprehensible to all transplant candidates in our catchment area (e.g., provide real-time translation services, offer in-person sessions, ensure educational materials meet our organization’s health literacy standards). Despite randomization, there appear to be differences between the study arms on sociodemographic characteristics. It is possible that sociodemographic factors including employment, gender, and ethnic background played a role in MuST AKT intervention completion. More research is needed to understand the interplay of these characteristics in program completion. We recruited participants without potential living donors who had not had a previous transplant and were either at the beginning of their transplant evaluation and/or were anticipated to have a long wait on the deceased donor waitlist determined by the transplant center. Those who consented to participate may have been particularly motivated to take the required steps to find a living kidney donor. Also, participants had pre-existing experience with virtual communication platforms prior to this study, which may have contributed to their willingness to participate in a virtual intervention. Satisfaction with the intervention was high, although a social desirability bias is possible for the acceptability questionnaire items, and only a small proportion of session 4 attendees completed interviews, which may not be representative of the patient’s entire social network. Still, the qualitative interviews were conducted by researchers independent of the study team to mitigate social desirability bias, and the findings suggest that the MuST AKT intervention was acceptable. Although the results may not generalize to other healthcare jurisdictions and populations, this initiative outlines an approach for personalizing LDKT interventions that may be useful to others. The self-efficacy assessments were not validated tools, but they adhere to the theoretical and measurement guidelines [31]. The intervention was primarily delivered by a behavioral scientist out of necessity due to the organization’s COVID-19 response, in which the social worker was unavailable to deliver the intervention. While these professions have different backgrounds and training, both individuals followed detailed intervention guides. In the definitive RCT, a social worker in kidney care will deliver the intervention, in which feasibility and acceptability will be re-evaluated. The sample size of the study was small but appropriate to evaluate feasibility. Results of statistical comparisons should be considered preliminary, and will be re-evaluated in a definitive RCT. While we did not set *a priori* cutoffs to evaluate feasibility, the comprehensiveness of the feasibility and acceptability metrics provide us with the required information to improve the intervention.

Overall, this study demonstrated that the MuST AKT intervention designed to engage transplant candidates and their social network to address barriers to LDKT, is acceptable and is feasible with minor modifications. A larger definitive RCT will be conducted to evaluate the optimized MuST AKT intervention and efficacy to increase LDKTs.

## Data Availability

The raw data supporting the conclusions of this article will be made available by the authors, without undue reservation.
