# Supplementary material for: The Multidisciplinary Support To Access living donor Kidney Transplant (MuST AKT) intervention: A Pilot Randomized Controlled Trial
Source: Transpl Int. 2026 Feb 25;39:15472. doi: 10.3389/ti.2026.15472 (PMC12975612; doi:10.3389/ti.2026.15472)
Supplement: Supplementary file 1 [file DataSheet2.pdf]

Supplementary Material: Appendix B

Interview Guide: Attendees of Session 4, Friends and Family

Preamble: The findings from this interview will be used to improve the MuST AKT program. Please be aware your decision to be or not be a living donor/advocate is confidential. The intention of this interview is not to sway your decision in either way.

Motivation for attending Session 4

1. What brought you to the Information Session on Kidney Disease and Living Kidney Donation?
2. What did you expect from attending the session?

Experience with MuST AKT Session 4 and Impacts (skills/capabilities, opportunities, motivation)

3. Please tell me about your experience attending this session.
4. What did you like about the session?
5. What did you not like about the session?
6. What information did you find most helpful? Informative?
7. Were there any topics that were covered that needed more time? Or less time?
8. Is there any information you felt was missing? Topics you would like to learn more about?
9. Did the session help you understand what it means to be an advocate for someone in need of a kidney transplant? What does being an advocate look like to you? Was anything clear/unclear about this in the sessions?
10. Did the session help you understand what it means to be a donor? What was clear/unclear about this in the session?
11. Did the session encourage you (or discourage you) to be an advocate? A donor? Do you think it would encourage/discourage others?

Before I ask these last questions, I want to make it very clear that I am asking this from the perspective of a researcher trying to understand how this program works and what could be done to improve it. I am not part of the program design team or the healthcare team, and there are no right or wrong answers. Your responses will be kept entirely confidential and will never be tied back to you or your family member/friend.

12. Did the session contribute to your decision to be or not be donor/advocate? [ask donor/advocate separately, as appropriate]
    a. Is there anything you would add to the session that might help you to make the best decision for you?
        i. If yes, what would you add?
    b. Do you intend to be a donor? [omit if already answered; reinforce there is no right or wrong answer and that they don't need to justify]

Wrap-up/Closure

1. Do you have any additional comments about your experience with the session that you think are important for the research team to know?
2. If we have additional questions for you, is it ok that we contact you again?
