# Supplementary material for: The Multidisciplinary Support To Access living donor Kidney Transplant (MuST AKT) intervention: A Pilot Randomized Controlled Trial
Source: Transpl Int. 2026 Feb 25;39:15472. doi: 10.3389/ti.2026.15472 (PMC12975612; doi:10.3389/ti.2026.15472)
Supplement: Supplementary file 2 [file DataSheet3.pdf]

Supplementary Material: Appendix C

Self-efficacy questions

1. On a scale of 0 to 100, with 0 being not at all confident and 100 being completely confident, how confident are you that you can talk to a friend or family member about being a potential living kidney donor?

0%	10%	20%	30%	40%	50%	60%	70%	80%	90%	100%

Not at all Confident — Completely Confident

2. On a scale of 0 to 100, with 0 being not at all confident and 100 being completely confident, how confident are you that you can find a living kidney donor?

0%	10%	20%	30%	40%	50%	60%	70%	80%	90%	100%

Not at all Confident — Completely Confident
