# Supplementary material for: The Multidisciplinary Support To Access living donor Kidney Transplant (MuST AKT) intervention: A Pilot Randomized Controlled Trial
Source: Transpl Int. 2026 Feb 25;39:15472. doi: 10.3389/ti.2026.15472 (PMC12975612; doi:10.3389/ti.2026.15472)
Supplement: Supplementary file 3 [file DataSheet1.pdf]

Supplementary Material: Appendix A

Interview Guide: MuST AKT Participants

Motivation for participating in MuST AKT

1. Can you tell me, what led you to take part in the MuST AKT study?
2. What did you expect from participating in the program? (what did you hope to get out of it?)
    a. Prompts: finding a donor, improved quality of life, having a sense of ownership over treatment options, learning more about living donation

Experience with MuST AKT and Impacts (skills/capabilities, opportunities, motivation)

3. Please tell me about your experience with the MuST AKT program.
4. What did you think of the content of the sessions?
    a. Prompt: easy/difficult to understand, enough/not enough content, too technical?
5. What was your experience with the MuST AKT facilitator?
    a. Prompts: what did you like about the facilitator, was there anything you didn't like
6. Was the MuST AKT program helpful in supporting you to identify potential donors and/or potential advocates?
    a. If yes, how did it help?
    b. If no, why didn't you find it helpful?
7. Did the MuST AKT program help you to communicate with potential donors and/or potential advocates?
    a. If yes, how so?
    b. If no, why not?
8. Since joining the MuST AKT study, have you shared your story with others?
    a. If yes: [quantify]
        i. How did you share your story? Please name all the ways you shared. Prompt: social media, in person, through an advocate, email, text, video, through community/sport groups. [quantify where?]
        ii. what was your experience with sharing your story?
        iii. Did you experience any challenges with sharing your story?
    b. If no: [quantify]
        i. Tell me about why sharing your story was not right for you.
        ii. Is there some way you could be supported to help you to share your story in the future?

Areas for Improvement

9. Is there anything you would change about the MuST AKT program?
    a. Prompts: Content, delivery method (zoom vs in-person), facilitator, # of sessions, frequency of sessions

10. Is there any information that you think could have been added to the sessions that could help identify or communicate with potential donors or advocates?
11. Did any of the topics require more or less time?

Overall Assessment of MuST AKT

12. Do you think the MuST AKT program will help you find a living kidney donor?
    a. Why? Why not?
13. Would you recommend this program to others?
    a. If yes, why?
    b. If no, why Not?

Wrap-up/Closure

14. That is all of our questions, is there anything else you would like to share with us about your experiences with the MuST AKT program?
15. If we have additional questions for you, is it ok that we contact you again?
